# Management of fluoroscopy-induced radiation ulcer: One-stage radical excision and immediate reconstruction

**DOI:** 10.1038/srep35875

**Published:** 2016-10-21

**Authors:** Kai-Che Wei, Kuo-Chung Yang, Lee-Wei Chen, Wen-Chung Liu, Wen-Chieh Chen, Wen-Yen Chiou, Ping-Chin Lai

**Affiliations:** 1 Department of Dermatology, Kaohsiung Veterans General Hospital, Taiwan; 2 Faculty of Yuhing Junior College of Health Care and Management, Kaohsiung, Taiwan; 3 Department of Plastic and Reconstructive Surgery, Kaohsiung Veterans General Hospital, Kaohsiung, Taiwan; 4 National Yang-Ming University School of Medicine, Taipei, Taiwan; 5 Department of Dermatology and Allergy, Technische Universität München, Munich, Germany; 6 IZZ-Immunologie Zentrum Zürich, Zürich, Switzerland; 7 Department of Radiation Oncology, Buddhist Dalin Tzu-Chi Hospital, Chiayi, Taiwan; 8 Department of Nephrology, Kidney Center, Chang Gung Memorial Hospital, Chang Gung School of Medicine, Chang Gung University, Linkou, Taiwan

## Abstract

With increasing use of cardiac fluoroscopic intervention, the incidence of fluoroscopy-induced radiation ulcer is increasing. Radiation ulcer is difficult to manage and currently there are no treatment guidelines. To identify the optimal treatment approaches for managing cardiac fluoroscopy-induced radiation ulcers, we retrospectively reviewed medical records of 13 patients with fluoroscopy-induced radiation ulcers receiving surgical interventions and following up in our hospital from 2012 to 2015. Conventional wound care and hyperbaric oxygen therapy were of little therapeutic benefit. Twelve patients received reconstruction with advancement flap or split thick skin graft. One-stage radical excision of radiation damaged area in eight cases with immediate reconstruction led to better outcomes than conservative excisions in four cases. Radical surgical excision to remove all the radiation damaged tissues in combination with immediate reconstruction appears to offer the optimal treatment results for cardiac fluoroscopy-induced radiation ulcers. Adequate excision of the damaged areas in both vertical (to the muscular fascia) and horizontal (beyond the sclerotic areas) dimension is pivotal to achieve good treatment outcomes.

Although guidelines for a safe radiation use during interventional procedures have been proposed^1^,^2^,^3^ and followed for years, cases with radiation injuries are still seen in the clinic. This may relate to the complexity or repetitive courses required to achieve acceptable cardiac outcomes. Recently, we noticed an increasing incidence of severe radiation dermatitis in our hospital^4^. Moreover, the trend is worldwide^5^,^6^,^7^,^8^,^9^,^10^,^11^,^12^,^13^.

The managements for skin damages resulting from radiotherapy and those from cardiac catheterization are very different. This is because the radiation beams used in these two settings are different in term of physics characteristics, energy levels, biological reactions and depth-dose distribution. Radiotherapy for cancer uses either beta ions or high-level energy photons at the energy level of mega-electron volts (MeV) or mega-volts (MV), while the radiation source for fluoroscopy is X-rays with a low energy level between 40 and 140 kilo-volts (KV). When a high-level radiation photon beam enters the body, it loses only little energy in the skin and subcutaneous tissue and deposits most of its energy at the deeper area (Fig. 1), whereas the energy of X-ray is absorbed within a few centimeters from the skin surface (Fig. 1). So far, few studies focused on the managements of fluoroscopic induced radiation ulcer. This study was aimed to identify the optimal treatment approaches for managing cardiac fluoroscopy-induced radiation ulcers by examining the impact of these approaches on the healing of radiation ulcer.

## Material and Methods

This retrospective study complied with the guidelines of the Declaration of Helsinki and was approved by the Medical Ethics Committee of Kaohsiung Veterans General Hospital, a tertiary referral center in southern Taiwan, with institutional Review Board approval (No. VGHKS15-CT3-08).

By reviewing medical records from 2012 to 2015 in our hospital, thirteen cases with radiation ulcers were identified. All these 13 patients required surgical interventions to treat their cardiac fluoroscopic related ulcers. Their demographic data, procedures received, skin changes, surgical interventions, treatment outcomes and the associated complications were recorded and analyzed (Table 1 and Supplement Table 1).

## Results

All of the 13 cases were male, and their mean age was 58.5 ± 12.4 years (range: 43 to 81 years). Eight of them had diabetes mellitus; but none of these diabetic cases had history of diabetic ulcers in limbs. None of these 13 cases had autoimmune disease or peripheral arterial occlusive disease. Twelve patients received percutaneous coronary interventions for coronary artery disease, and one patient underwent electrophysiological ablation for a refractory supraventricular accessory pathway. Notably, fluoroscopic interventions were performed at least three times in all patients. The estimated accumulative peak skin dose is 19.1 ± 5.3 Grays (group A, range: 14.3 to 28.8) and 24.7 ± 7.1 Gy (Group B and C, range: 17.8 to 40.1) (Supplement Table 1). The radiation ulcers were found in right subscapular and inner arm areas in 12 cases, whereas one had his lesion at the left lower back. All patients showed typical manifestations of fluoroscopy-induced radiation ulcer, with a sharply-demarcated rectangle or square patch and central ulceration. The most common symptom of these patients was intolerable pain. At the first presentation to the surgical clinic, the severity of ulcers of each patient had been all graded as stage 4 based on CTCAE v4.03 Grade Severity Score.

All cases had non-healing ulcers, which often worsened with conventional wound care, for at least 3 months. Five cases received hyperbaric oxygen therapy before surgery but no therapeutic effects were noted. One case received vacuum-assisted wound closure and hyperbaric oxygen therapy after repetitive conservative debridement. Although his wound became smaller, it failed to heal even after following up for 1 year. Like other cardiac diseases patients, repetitive anesthesia and wound debridement increase the risk of cardiac attack in these patients. One of our patients indeed developed unstable angina during debridement and need further cardiac fluoroscopic interventions to control his cardiac ischemia.

We categorized our patients into three groups according to their treatment modalities (Table 1): Group A: conservative debridement with/without reconstruction flap at later stage; Group B: radical debridement plus immediate reconstruction with local advancement flap; Group C: radical debridement plus immediate reconstruction with split-thickness skin grafting (STSG).

### Group A (Conservative debridement with/without reconstruction flap at later stage)

The first five cases (case 1 to case 5) underwent three to five sessions of conservative debridement (Fig. 2), aiming to remove only the necrotic ulcerated area with minimal tissue destruction of the surrounding sclerotic area. We found that these wounds inevitably continued to worsen after this approach. Reconstruction with localized rotation flaps were performed at the later stage in four cases, however, all these reconstruction flaps failed.

### Group B (Radical excision plus immediate local advancement flap)

Patients in this group (case 6 to case 10), the debridement (excision) margins were extended laterally to the outer zone (exceeding the white sclerotic area) of damaged skin and vertically deep to the muscular fascia layer (Figs 3 and 4). Reconstruction with a rotation flap (Fig. 3) or local advancement fasciocutaneous flap (Fig. 4) was performed at the same time. All cases had immediate pain relief and achieved good wound healing without major complications.

### Group C (Radical excision plus immediate STSG)

Patients in this group (case 11 to 13) received radical excision (the same excision method as group B) and immediate coverage with split thickness skin graft (Fig. 5). All three patients achieved good wound healing within 4 weeks without obvious complications.

## Discussion

The aims of treating radiation ulcers include preventing infection, controlling pain, closing wounds and preventing malignancy. When planning an operation, clinicians should bear in mind that the zone of radiation injury is larger than the area exhibiting clinical symptoms and signs, and inadequate excision inevitably leads to treatment failure. In addition, the residual radiation damaged tissue may increase the risk of subsequent radiation-related malignancies.

As a general rule, the severity of radiation injury increases with accumulated exposure dose i.e. if the exposed dose is less than a threshold, the radiation dermatitis is typically not clinically detectable but once the accumulated exposure dose exceeds the threshold, radiation dermatitis occurs. To recover from the injury, the damaged tissues need stem cells either from radiated area or adjacent tissues. If enough number of stem cells still present, the lesion will usually respond to the conventional wound care. On the other hand, when a radiation ulcer appears, the adjacent tissues tend to suffer severe radiation damage as well. As a result, these tissues not only have limit ability to facilitate lesion site’s recovery but also impose further stresses on healing processes. Therefore, complete removal of these severely damaged tissues is the cornerstone of radiation ulcers managements. From our experience, this principle applies to all the radiation ulcers treatments, irrespective of the accumulated exposure dose.

Another possible cause of radiation ulcer is radiation vasculopathy^14^, which leads to vessel occlusion and tissue ischemia in the irradiated region. This will also jeopardize the wound healing processes and result in poor adhesion of the reconstruction flap. Radiation vasculopathy is also the underlying reason why these patients frequently experience severe intolerable pain, which is of similar characteristics as ischemic pain, and why reconstruction flaps frequently fail to heal the radiation ulcer.

**Figure 1:**
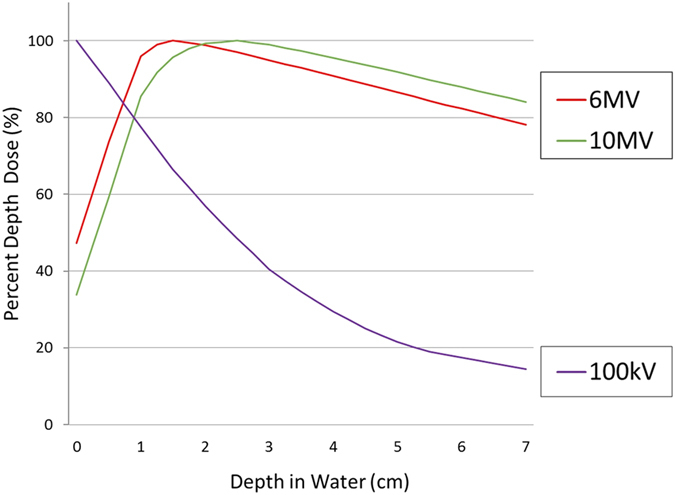
Depth dose curves in water or soft tissues for various quantities of radiation beams. *Line red:* linear accelerator 6 MV photon beam. *Line green*: linear accelerator 10 MV photon beam. *Line purple*: 100 kV X-rays filtered with HVL = 2 mm Al.

**Figure 2:**
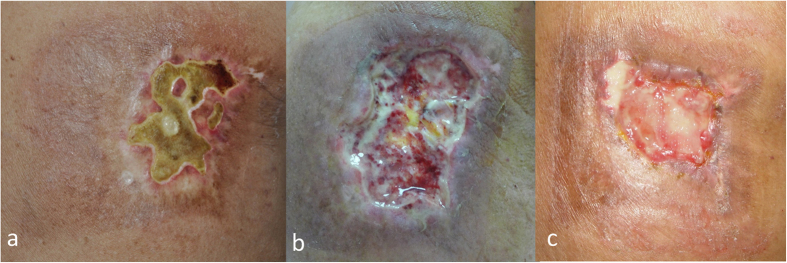
Group A: Conservative debridement with/without reconstruction flap at later stage. (**a**) An 81-year-old man (case 1) had a painful sharply-demarcated square-shaped patch with a centrally located thick eschar (4.5 × 2.5 cm) and surrounding fibrosis for 5 months on his right sub-scapular area. This lesion appeared 2 months after per-cutaneous coronary intervention. (**b**) After three attempts of conservative debridement (only of the ulcerated area) and regular wound care within two months, the ulcer was clean but not healed. (**c**) After regular hyperbaric oxygen therapy and wound care with vacuum-assisted closure for 6 months, the ulcer became smaller but still did not heal.

**Figure 3:**
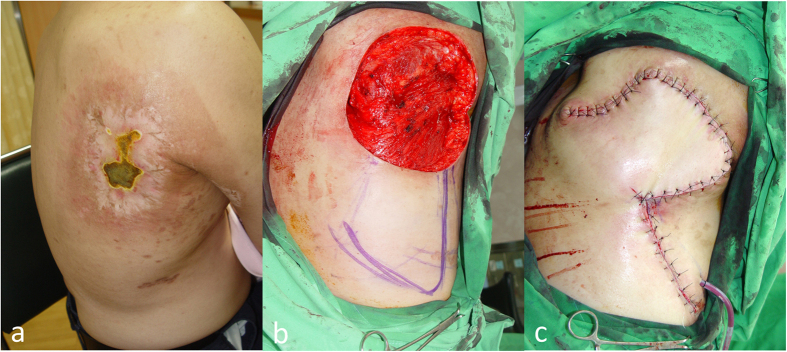
Group B: Radical excision and immediate reconstruction of rotation flap. (**a**) A 52-year-old man (case 6) presented with a typical fluoroscopic radiation-induced skin lesion as a target-like plaque with three differently-colored rims and central ulceration. (**b**) Radical excision of necrotic and sclerotic areas with a deep margin to muscular fascia layer was performed. (**c**) Immediate reconstruction with local rotation fasciocutaneous flap was done. Wound healed smoothly one month after surgery. No recurrence was noted in the following 15 months.

**Figure 4:**
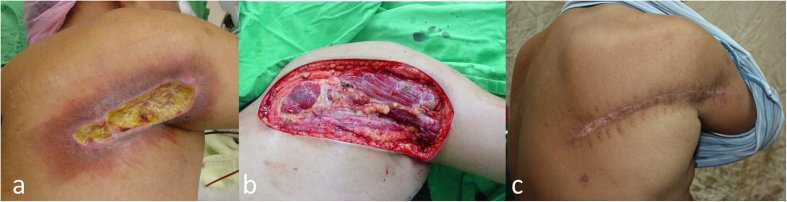
Group B: Radical excision and immediate local advancement flap. (**a**) A 52-year-old man (case 7) had a deep necrotic ulcer with erythematous-to-brownish patch in the periphery for 10 months on his right subscapular and arm region. The lesion occurred 2 months after percutaneous coronary intervention. (**b**) Radical excision of necrotic and sclerotic areas with a deep margin to muscular fascia layer was performed. (**c**) Local advancement flap was performed and wound healed two weeks later. No recurrence was noted in the following 14 months.

**Figure 5:**
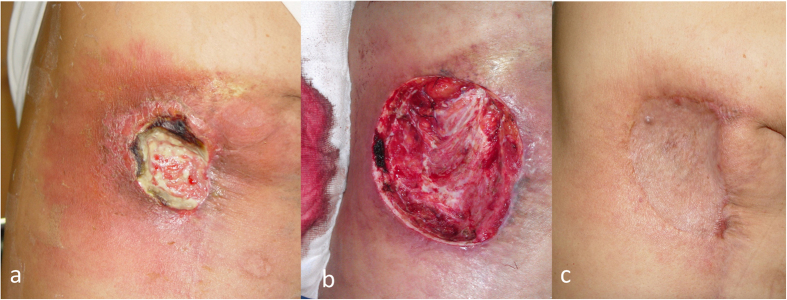
Group C: Radical excision and immediate split thick skin graft (STSG). (**a**) A 70-year-old man (case 11) presented with a large deep necrotic ulcer with bacterial infection and pus formation, surrounded by a square-shaped erythematous sclerotic plaque for 6 months on his right lateral back. The lesion was found 1 month after electrophysiological ablation of cardiac accessory pathway. (**b**) Excision of all the sclerotic areas deep to the muscular fascia layer was done with immediate coverage with STSG. (**c**) Following up at 16^th^ month in the clinic, good uptake of STSG was noted without recurrence.

Cardiac fluoroscopic procedures adopt the radiation energy levels at about 100 kV. Thus, most of the fluoroscopy-induced radiation damages are located within the superficial layers of skin and their severity decrease steadily down to deeper area (Supplement Fig. 1). A typical lesion of a fluoroscopy-induced radiation ulcer is a rectangle or square lesion with three concentric zones in different colors. The shape of the lesion matches the fluoroscopic exposure field, and the color distribution correlates with the radiation dose that was applied (Fig. 3a). Necrotic tissue, the center of the lesion, has the highest radiation dose, whereas the outermost pigmented dry-surface “flame burn-like” zone has the lowest radiation dose. The middle layer, the white sclerotic telangiectasia zone, has a medium dose of radiation. The sclerosis indicates loss of adnexal structures where follicular stem cells resides in. Thus, radiation wound in the sclerotic zone is difficult to heal. Based on these, the horizontal excision margin should aim to remove the sclerotic zone as we did in group B and C patients. However, to decide the vertical excision depth is more challenging. Theoretically, the most severely damaged zone is within the uppermost 2 cm below the skin surface (Supplement Fig. 1). Hence, the depth of excision should be at least 2 cm. On top of this, excision as deep as presence of viable healthy-looking tissue is also critical. Nonetheless, in our experience, none of the excision depths exceeded muscular layer.

To achieve primary wound healing, reconstructive methods are usually necessary. However, it is not known whether a vascularized flap is better than a simple skin grafting in bringing wound healing in these patients^15^,^16^. Our study showed that there are no differences in the outcomes of group B patients (radical excision plus vascularized fasciocutaneous flap) and group C patients (radical excision plus STSG). Our results support the concept that an adequate excision, independent of the methods of reconstruction, is the critical point for treating radiation ulcers.

## Conclusion

In the management of fluoroscopy-induced chronic radiation ulcers, one-stage radical excision with immediate wound reconstruction showed the best result in our study. It alleviates patients’ suffering and the risk of repetitive anesthesia. Adequate excision of the radiation damaged areas including the vertical (to the viable healthier muscular fascia) and the horizontal (beyond the sclerotic and telangiectasia area) dimensions is the key to achieve good treatment outcomes.

## Limitation

This study focuses on the most severe radiation injuries (radiation ulcers), thus our findings can not be applied to the management of radiation dermatitis. Although the underlying diseases of these patients were diverse, none of them had history of diabetic ulcer, or peripheral arterial occlusive disease or autoimmune disease.

## Additional Information

**How to cite this article**: Wei, K.-C. *et al*. Management of fluoroscopy-induced radiation ulcer: One-stage radical excision and immediate reconstruction. *Sci. Rep.* **6**, 35875; doi: 10.1038/srep35875 (2016).

## Supplementary Material

Supplementary Information

## Figures and Tables

**Table 1:** The summary of patients’ characteristics and demographic data.

Patient number	Excision Group		Sex	Age	Co-morbidities	Radiation Exposure	Surgical procedures & Outcome				
	Conservative excision (CE) versus Radical excision (RE)					Numbers of Procedures	Reconstruction	Outcome	Time to Complete Heal	Complications	Follow-up Time **
1	CE		M	81	HTN, Dyslipidemia, CKD (Cr 2.2)	5	ND	Not healed	NA	Unstable angina	22 months
2			M	82	DM, HTN, CHF, CKD (Cr 3.1)	3	Local flap X 1 wound closure X 1	Healed	4 months	Wound dehiscence for many times	15 months
3			M	45	DM, HTN, Dyslipidemia	3	Rotation flap X 2 wound closure X 1	Healed	6 months	Poor healing and flap necrosis in the wound edge	16 months
4			M	62	DM, HTN, Liver cirrhosis	3	Rotation flap X 2	Not healed	NA	Poor healing and necrosis of flap	15 months
5			M	52	DM, HTN	3	Local flap X 2 wound closure X 2	Healed	5 months	Wound dehiscence for many times	5 months
6	RE	+Flap	M	52	DM, Dyslipidemia	3	Rotation flap X 1	Healed	2 weeks	Nil	15 months
7			M	52	DM, HTN, Dyslipidemia	4	Local flap X 1	Healed	3 weeks	Nil	14 months
8			M	60	DM, HTN, Dyslipidemia	3	Local flap X 1	Healed	2 weeks	Nil	13 months
9			M	43	DM, HTN, Dyslipidemia	3	Local flap X 1	Healed	2 weeks	Nil	15 months
10			M	50	HTN, Dyslipidemia, Hyperuricemia	3	Rotation flap X 1	Healed	3 weeks	Nil	7 months
11	RE	+STSG	M	70	HTN*	3	STSG X 1	Healed	4 weeks	Nil	21 months
12			M	55	HTN	3	STSG X 1	Healed	2 weeks	Nil	29 months
13			M	57	HTN, Dyslipidemia	4	STSG X 1	Healed	2 weeks	Nil	8 months

NA: Not applicable; ND: Not done.

CHF: Congestive heart failure; CKD: Chronic kidney disease; DM: Diabetes mellitus; HTN: Hypertension.

STSG: Split thickness skin graft.

*This 70 year-old man received elctrophysiologic ablation for PSVT/aVRT, not percutaneous coronary intervention.

**Follow-up time is calculated since after the latest operation.
