# Supplementary material for: Management of fluoroscopy-induced radiation ulcer: One-stage radical excision and immediate reconstruction
Source: Sci Rep. 2016 Oct 21;6:35875. doi: 10.1038/srep35875 (PMC5073289; doi:10.1038/srep35875)
Supplement: Supplementary Information [file srep35875-s1.pdf]

Management of fluoroscopy-induced radiation ulcer: One-stage radical excision and immediate reconstruction

Authors:

Kai-Che WEI MD [1,2], Kuo-Chung YANG MD [3,4], Lee-Wei CHEN MD PhD [3,4], Wen-Chung LIU MD [3,4], Wen-Chieh CHEN MD [5,6], Wen-Yen CHIOU MD [7], Ping-Chin LAI MD PhD [8]

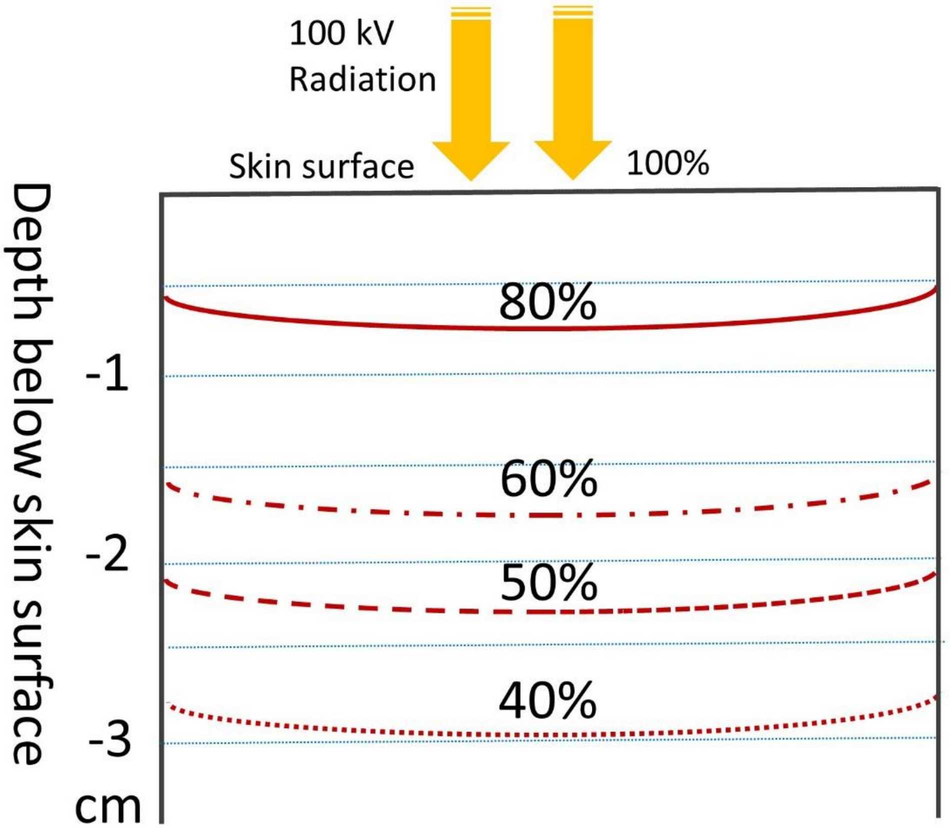

Supplement Figure 1

The relative radiation dose in different depth below skin surface

Patient number	Conservative excision (CE) versus Radical excision (RE)		Estimated peak skin dose (Gy)	CTCAE v4.03 Grade Severity	Pre-op ulcer size (cm) (length x width)	Excision size* (cm) (length x width x depth)
1	CE		17.8	4	8 x 6	8x6.2x1.4
2			14.6	4	3 x 2	3.2 x 2.2 x 1.2
3			28.8	4	3 x 2	3.5 x 3.2 x 0.8
4			14.3	4	2.5 x 2	3 x2 x 1.5
5			19.9	4	4 x 4	4.2 x 4 x 1.5
6	RE	+ Flap	40.1	4	5 x 3	12 x 8.5 x 3.5
7			23.8	4	8 x 7	14 x 13 x 3.2
8			17.8	4	10 x 3	14.8 x 6 x 3.2
9			19.0	4	12 x 5	18.6 x 6 x 3
10			28.6	4	7.5 x 6.6	8.5 x 7.7 x 2
11	RE	+ STSG	NA	4	6 x 5 x 1	9 x 7 x 3
12			22.9	4	6 x 4	8 x 6 x 2
13			20.5	4	5 x 4	17.5 x 10.5 x 2

NA: Not available;

* The information was obtained from the operation notes and histology reports.

Supplement Table 1

The estimated peak skin radiation doses, severity of ulcer and detailed surgery area with precise size information of each patient
